# Neurocognitive deficits in depression: a systematic review of cognitive impairment in the acute and remitted state

**DOI:** 10.1007/s00406-022-01479-5

**Published:** 2022-09-01

**Authors:** Dominik Kriesche, Christian F. J. Woll, Nadja Tschentscher, Rolf R. Engel, Susanne Karch

**Affiliations:** 1 Department of Psychiatry and Psychotherapy, Section for Clinical Psychology and Psychophysiology, Hospital of the University of Munich, Nußbaumstr. 7, 80336 Munich, Germany; 2 Department of Psychology and Educational Sciences, Clinical Psychology of Children and Adolescents, Leopoldstr. 13, 80802 Munich, Germany

**Keywords:** Depression, Neuropsychology, Cognition, Deficits, Impairment

## Abstract

Previous research suggests a broad range of deficits in major depressive disorder. Our goal was to update the current assumptions and investigate the extent of cognitive impairment in depression in the acute and remitted state. A systematic review of the existing literature between 2009 and 2019 assessing the risk of bias within the included studies was performed. Of the 42 articles reviewed, an unclear risk of bias was shown overall. The risk of bias mainly concerned the sample selection, inadequate remedial measures, as well as the lack of blinding the assessors. In the acute phase, we found strong support for impairment in processing speed, learning, and memory. Follow-up studies and direct comparisons revealed less pronounced deficits in remission, however, deficits were still present in attention, learning and memory, and working memory. A positive correlation between the number of episodes and cognitive deficits as well as depression severity and cognitive deficits was reported. The results also demonstrate a resemblance between the cognitive profiles in bipolar disorder and depression. Comparisons of depression with schizophrenia led to unclear results, at times suggesting an overlap in cognitive performance. The main findings support the global deficit hypothesis and align with results from prior meta-analyses and reviews. Recommendations for future research are also presented.

## Introduction

Cognitive dysfunction is considered one of the core symptoms of depression in the current diagnostic and statistical manual of mental disorders [1]. It is described as a reduced ability to think, concentrate, or make decisions. The cognitive level of performance in patients with major depressive disorder (MDD) is of high practical relevance because cognitive deficits are associated with a lowered ability to function in everyday life, reduced psychotherapeutic treatment success, and increased suicidality [2, 3].

Previous research has shown deficits in the areas of executive functions (EF), memory, psychomotor speed, and attention in patients with MDD as compared to healthy controls (HC) [4, 5]. These deficits range from mild to severe and can partially persist after remission from depression [4–6]. Hammar and Ardal [4] summarize deficits in attention, EF, and memory after remission. They point out that a reduction of depressive symptoms does not necessarily lead to cognitive improvement. This could be due to cognitive impairments being trait markers rather than state markers of depression [7]. So far, research on the connection between symptom severity and cognitive impairment has been inconsistent, but overall, a tendency for a positive correlation is reported [8, 9]. Besides these studies, systematic reviews and meta-analyses comparing the evidence of cognitive impairment in depression with other disorders are rather rare. Bora et al. [10] state that there is only a small difference in cognitive functioning between schizophrenia (SCH) and affective psychoses, despite the popular belief that these are two qualitatively distinguishable disorders. The authors revealed that, among other factors, more severe negative symptoms moderated the negative effect on cognitive functions. Similar results are reported by Stefanopoulou et al. [11] who also found quantitative rather than qualitative differences comparing MDD, bipolar disorder (BD), and SCH. The cognitive profiles in euthymic BD patients point to similar results as shown in remitted depressed (RD) patients [12].

In our systematic review, we aim to summarize the research findings of the last 10 years on neuropsychological deficits of adults suffering from MDD. Based on the latest research on cognitive deficits in MDD described above, we expect (1) homogeneous results concerning the impaired areas: EF, memory, attention, and psychomotor speed. Regarding the course of the deficits, we hypothesize (2) only a partially restored cognitive performance profile after remission. In addition, we assume (3) a positive correlation between the number of episodes and cognitive impairment as well as (4) a positive correlation between severity of depression and cognitive impairment. Additionally, we will summarize results considering the frequency of cognitive deficits in depression and differences between MDD and BD as well as MDD and SCH.

## Methods

To guarantee a transparent and reproducible research process [13], we (a) disclosed all systematic review data, including risk of bias coding, on the Open Science Framework (OSF; see https://osf.io/hn3w8/), (b) adhered to the PRISMA 2020 reporting guidelines [14], (c) pre-registered our introduction and method section on the Open Science Framework before starting with data collection (see https://osf.io/5by6j), (d) hereby allow other researchers to re-analyze our data including our entire literature hits from databases in common file formats, and (e) recruited expertise.

## Inclusion criteria

We included studies published in English examining the neuropsychological functions of at least 18-year-old participants who received a depressive disorder diagnosis according to international diagnostic manuals (e.g., ICD-10, DSM-IV, and DSM-5). The selected studies measured cognitive functions by reliable, valid, and objective neuropsychological tests. The test data could reflect the current status, a follow-up (e.g., 1 year after onset of illness), or compare cognitive deficits with other diseases.

Consequently, we excluded studies on animals, biological studies that aim at identifying disorder-specific genes, and studies in which the participants mainly suffer from comorbid psychological diseases (e.g., dementia, addiction). Furthermore, no family studies or research with a focus on the effects of interventions (e.g., therapeutic effects) were considered. We did not include studies examining social cognitions (e.g., perspective taking, empathy) or studies with participants not meeting depression criteria.

## Information sources and search

We chose the online databases PsycINFO, Scopus, and PubMed for our literature search. The search term was created by adapting search terms of already conducted reviews. Our search term was applied to the titles of the primary studies. For all databases, we used the following search term: “(depress*) AND (cogniti* OR neuropsychological) AND (impairment* OR function* OR deficit*)”. For PsycINFO we deactivated the option “linked full text”, set the publication year to 2009–2019, set the publication type to “Peer-reviewed Journal”, and activated the box “English”. In Scopus, we set the date range to 2009–2019, set the document type to “article”, and access type to “All”. The only filter activated in PubMed was restricting the search to studies published within the last ten years. Studies published until the 1st of October 2019 were included.

## Study selection and data collection

Three members of our research team were responsible for the selection process. Non-relevant studies were excluded and assigned to different categories according to why they were rejected. On the other hand, studies that appeared relevant were downloaded in a RIS-format and saved in Citavi. The main author (DK) double-checked the excluded and included studies. For included studies, we extracted data for the following variables: author names, publication year, date and place of the study, diagnosis and age of patients, applied psychological tests, and the outcome of the tests.

## Outcome measures

To compare the results of different studies, we used statistical values of reliable and valid standardized neuropsychological tests. All reported differences in our review were based on statistically significant results. We did not rely on descriptive evaluations (e.g., “better”, “higher scores”).

## Assessment of the risk of bias in individual trials

To assess the risk of bias in individual studies, we used and adapted the Cochrane risk of bias tool for randomized-controlled trials [15]. We omitted the items assessing random sequence generation and allocation concealment because they only apply to randomized-controlled trials. Instead, we assessed selection bias and verified if a clear and thorough diagnostic procedure was applied. A detailed description and explanation of our items as well as citations of the primary studies to support our evaluation is provided in our excel coding sheet on the OSF (see https://osf.io/hn3w8/). We summarized the assessment of risk of bias within and across trials primarily by following an example by Higgins et al. [15] (see Table 1).

**Table 1:** Summary assessments of risk of bias within and across studies (adapted from Higgins et al. [15])

Risk of bias	Interpretation	Within trial	Across trials
Low risk of bias	Bias, if present, is unlikely to alter the results seriously	Low risk of bias for all key items	The majority of trials carry a low risk of bias
Unclear risk of bias	A risk of bias raises some doubt about the results	Low or unclear risk of bias for all key items	The majority of trials carry a low or unclear risk of bias
High risk of bias	Bias may alter the results seriously	High risk of bias for one or more key items	The majority of trials carry a high risk of bias

## Results

### Included studies

**Fig. 1:**
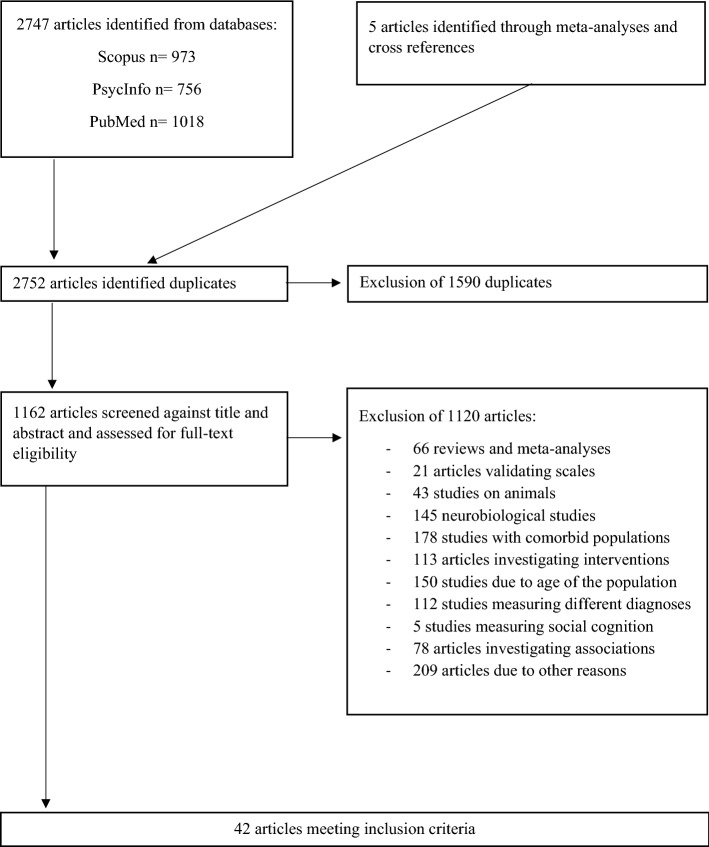
Flowchart of the selection process (adapted from Page et al.[14])

A total of 1162 articles were screened for eligibility. After exclusion of 1120, we included a total of 42 studies [16–57]. Figure 1 illustrates our search, screening, and selection process.

For example, a study by Eraydin et al. [58] was excluded because the depression diagnostics were conducted through an online tool which limited the reliability. Likewise, we excluded a study by Ambaw et al. [59] which used only one cognitive screening test (MMSE), that had not been validated in the study’s country and applied unclear inclusion criteria.

## Risk of bias

Based on the modified Cochrane Risk of Bias Tool, ten studies carried a high risk of bias (see Table 2). Eight of these studies showed methodological deficits in the selection process, one study reported not blinding the assessors, and one study did not address incomplete outcome data. A closer look of the studies showing a weaker selection process revealed differences in the characteristics of the experimental and the control group. These sample differences, which were not adequately corrected, led to comparisons of heterogeneous groups. The remaining studies showed an unclear risk of bias, mainly due to the absence of any blinding measurements.

**Table 2:** Summary assessment of the risk of bias within studies by applying the Cochrane Risk of Bias Tool (Higgins et al. [15])

Author, year	Selection bias	Clear diagnostics	Blinding (patients)	Detection bias	Incomplete outcome data addressed	Free of selective reporting	Summary assessment
Albert [16]	?	+	?	?	?	+	**?**
Ardal [17]	+	?	?	?	+	+	**?**
Baune [18]	?	+	?	?	?	+	**?**
Bhardwaj [19]	+	+	?	?	+	+	**?**
Boeker [20]	?	?	?	?	?	+	**?**
Castaneda [21]	?	?	?	+	+	+	**?**
Constant [22]	–	?	?	?	+	+	**–**
Daniel [23]	?	?	?	+	+	+	**?**
Gooren [24]	?	?	+	?	?	+	**?**
Gruber [25]	+	?	?	?	?	+	**?**
Grützner [26]	+	+	?	?	+	?	**?**
Halvorsen [27]	?	+	?	?	+	+	**?**
Hammar [28]	+	?	?	?	+	+	**?**
Hasselbalch [29]	?	?	?	+	+	+	**?**
Hsu [30]	+	?	?	?	+	+	**?**
Jia [31]	?	?	?	?	+	+	**?**
Kaygusuz [32]	–	+	?	?	+	?	**–**
Keilp [33]	?	?	?	?	?	+	**?**
Leposavic [34]	?	?	?	?	?	+	**?**
Liu [35]	?	+	?	?	?	+	**?**
Lyche [36]	–	+	?	?	+	+	**–**
Lyche [37]	–	+	?	?	+	+	**–**
Maalouf [38]	+	+	?	?	+	+	**?**
Mak [39]	?	+	?	?	+	?	**?**
McClintock [40]	–	+	+	+	?	+	**–**
Moniz [41]	?	?	?	?	?	+	**?**
Neu [42]	–	+	?	?	+	+	**–**
Peters [43]	?	+	?	?	+	+	**?**
Preiss [44]	+	+	?	–	+	+	**–**
Preiss [45]	+	+	?	?	+	+	**?**
Rampacher [46]	+	+	?	?	+	+	**?**
Reppermund [47]	+	+	?	?	-	?	**–**
Roca [48]	?	+	?	?	+	+	**?**
Schaub [49]	?	?	?	?	+	+	**?**
Schmid [50]	+	+	?	?	?	+	**?**
Schulze [51]	?	?	?	?	+	+	**?**
Schwert [52]	?	+	?	?	+	+	**?**
Sostaric [53]	+	?	?	?	+	+	**?**
Taconnat [54]	+	?	?	?	?	+	**?**
Talarowska [55]	–	?	?	?	?	+	**–**
Wekking [56]	–	?	?	?	?	?	**–**
Zaremba [57]	+	?	?	?	?	+	**?**

Bold print represents the summary assessment

Notes + : represents a low risk of bias; ?: represents an unclear risk of bias; -: represents a high risk of bias

Overall, our set of included studies carries an unclear risk of bias (see Table 1). Therefore, the results and conclusions of this review must be interpreted with caution.

## Currently depressed (CD) patients vs. HC group

### Information processing speed

The vast majority of studies demonstrated a significant reduction of information processing speed in CD patients [16, 24, 35, 36, 39, 41, 42, 47, 51, 52, 54, 57]. Three studies did not show significant differences [27, 28, 30]. An exemplary overview of assignments of tests to cognitive functions is presented in Table 3. Table 4 shows all the included studies for the comparison between CD and HC.

**Table 3:** Assignments of tests to neuropsychological functions

Cognitive function	Cognitive sub-function	Test
Attention	Alertness	CalCAP, COGBAT Alertness, TEA phasic alertness task
	Divided attention	COGBAT: Divided Attention, TAP divided attention
	Sustained attention	Continuous performance task (CPT)
Executive function	Cognitive flexibility	CANTAB intra-extradimensional set shift (IED), TMT B, Wisconsin Card Sorting Test (WCST)
	Inhibition	D-KEFS Color–Word Interference Test, Stroop test
	Planning	Tower test
Information processing speed		CANTAB rapid visual information processing (RVIP), TMT A
Learning and memory	Verbal	Rey‘s Auditory Verbal Learning Test, Wechsler Memory Scale (WMS), Word memory task (WMT), CANTAB paired associates learning (PAL)
	Visual	Benton Visual Retention Test, Doors test, Wechsler Memory Scale (WMS),
Verbal fluency		Animal naming, Controlled Oral Word Association Test (COWA), D-KEFS Verbal Fluency
Visuospatial ability		RBANS visuospatial ability
Working memory		Digit span, Paced Auditory Serial Addition Test (PASAT)

*D-KEFS* Delis-Kaplan Executive Function System, *CalCAP* California Computerized Assessment Package, *CANTAB* Cambridge Neuropsychological Test Automated Battery, *COGBAT* Cognitive Basic Assessment, *RBANS* Repeatable Battery for the Assessment of Neuropsychological Status, *TAP* Testbatterie zur Aufmerksamkeitsprüfung, *TEA* Test of Everyday Attention, *TMT* Trail Making Test

**Table 4:** Characteristics and main results of studies investigating currently depressed samples

Author & year	Currently depressed group					Healthy control group				Cognitive tests		Main results
	*n* (% female)	Age	Characteristics	Symptom severity		*n* (% female)	Age	Symptom severity				
Albert [16]	91 (66.3)	35.9, 9.0	Recurrent MDD, IP	24.0, 4.4 (MADRS)		105 (64.8)	30.2, 9.1	0.7, 1.1 (MADRS)		Logical Memory 1 & 2, Benton Visual Retention Test, RVLT, COWA, TMT A + B, Animal Naming, Stroop Color, Symbol-Digit Modality, Digit Span Forward & Backward		D < HC: processing speed D = HC: WM, visual memory, EF
Ardal [17]	19 (52.6)	42.5, 10	Acute unipolar MDD, IP and OP	22.2, 3.6 (HDRS)		19 (52.6)	42, 9.7	–		Stroop test (Hugdahl version)		D < HC: inhibition
Baune [18]	26 (missing / wrong)	46.0, 12.1	MDD, OP	18.0, 5.9 (HAMD-D 17)		206 (61.2)	47.5, 15.2	–		RBANS		D < HC: immediate memory, visuospatial/construction, language, attention, D = HC: delayed memory
Boeker [20]	28 (46.4)	39.7, 11.4	MDD, IP	25.9, 8.2 (BDI); 28.5, 7.0 (HDRS-21)		28 (46.4)	35.0 (SD error)	–		CANTAB		D < HC: visual learning, memory, WM, EF, sustained attention
Constant [22]	25 (64.0)	45.8, 11.0	MDD, OP	25.6, 8.6 (BDI); 22.0, 5.8 (GDS)		29 (62.1)	47.5, 12.6	2.4, 3.2 (BDI), 4.0, 3.3 (GDS)		TEA 1.5, PASAT, Word memory test, Doors test		D < HC: alertness, WM, verbal and visual memory
Gooren [24]	102 (72)	52.4, 11.9	MDD, IP	19.7, 4.7 (MES)		85 (68.2)	52.6, 68.2	–		RAVLT, TMT A, Verbal Fluency Test, WMS		D < HC: verbal learning, verbal memory, processing speed, verbal fluency, visual memory
Gruber [25]	18 (78)	46.5, 10.7	CD, IP	4.3, 1.0 (CGI); 19.9, 10.7 (BDI); 20.1, 10.7 (MADRS)		18 (78)	44.6, 11.6	–		Computer-based behavioral experiment measuring process- and circuit-specific WM tasks		D < HC: articulatory rehearsal component of verbal WM D = HC: non-articulatory maintenance of phonological information, visuospatial WM
Halvorsen [27]	37 (73)	37.5, 12.0	CD, OP	25.3, 9.2 (BDI-II)		50 (78)	38, 12.7	3.1, 2.9 (BDI-II)		D-KEFS: Color-Word and Verbal Fluency, WCST-64, TMT A + B, calCAP, Halstead-Reitan Seashore Rhythm Test, WAIS-III: digit span forward, digit span backward, digit symbol coding,		D < HC: WM, processing speed D = HC: EF, attention, verbal fluency
Hammar [28]	24 (75)	38.1, 11.4	Recurrent MDD, acute, IP	22.4, 4.5 (HDRS); 27.1, 5.2 (MADRS)		24 (75)	37.1, 11.5	–		D-KEFS: TMT, Colour-Word Interference Test, Verbal Fluency Test, Tower Test		D < HC: inhibition, inhibition/switching, category fluency, color naming D = HC: processing speed, cognitive flexibility, planning, word reading, letter fluency, category switching
Hsu [30]	26 (76.9)	23.1, 6.2	CD, OP	23.2, 10.2 (BDI-II)		29 (62)	24.1, 6.8	5.9, 7.9 (BDI-II)		D-KEFS: Color-Word interference test, TMT A + B, Emotional Stroop Task		D < HC: selective attention, inhibition D = HC: psychomotor speed, cognitive flexibility/set shifting
Jia [31]	62 (52.6)	35.6, 12.7	FE drug naïve depressed OP	–		90 (66.7)	35, 10.7	–		RBANS		FED < HC: language, delayed memory FED = HC: Immediate memory, attention, visuoconstruction
Jia [31]	111 (65.8)	41.6, 12.1	Medicated FE unipolar depressed IP	–		90 (66.7)	35, 10.7	–		RBANS		MD < HC: immediate memory, delayed memory, language, MD = HC: attention, visuoconstruction
Liu [35]	30 (66.7)	27.8, 7.2	MDD, IP and OP	24.1, 4.5 (HDRS-24)		30 (53.3)	24.5, 3.0	–		WAIS-RC: digit symbol-coding, digit span; WMS-RC; modified WCST, TMT-B, VFT; modified Stroop Color Word Test:		D < HC: psychomotor speed, WM, visual memory, attention switching, verbal fluency D = HC: attention, cognitive flexibility, response inhibition
Lyche [36]	37 (62.2)	44.2, 12.3	MDD without anxiety	21.4, 11.1 (BDI)		91 (69.3)	35.8, 12.0	2.1, 2.7 (BDI)		WAIS-III: picture completion, similarities; CANTAB: intra-extra dimensional, spatial WM, stop signal task		D < HC: psychomotor speed D = HC: set shifting, WM, inhibition
Lyche [37]	37 (62.2)	44.2, 12.3	MDD	21.3, 11.1 (BDI)		92 (68.5)	35.7, 12.0	2.1, 2.7 (BDI)		D-KEFS: Color Word Interference Test, Attentional Network Test		D < HC: alertness D = HC: switching/inhibition, inhibition, EF
Mak 2018	35 (57.1)	24.9, 4.4	MDD	23.2, 5.4 (MADRS)		35 (65.7)	22.9, 3.2	0.2, 0.4 (MADRS)		TMT, Digit Span, WCST, Category fluency test, Chinese AVLT, WMS		D < HC: processing speed, cognitive flexibility D = HC: attention switching, WM, verbal fluency, verbal and visual memory
Maloof [38]	20 (80)	34.2, 9.4	acute recurrent unipolar depressed, OP	24.8, 5.8 (HAM-D)		28 (67.9)	31.9, 9.4	–		CANTAB: Rapid Visual Processing (RVP), Stockings of Cambridge (SOC), Delayed matching to Sample task (DMS)		D < HC: EF D = HC: sustained attention, memory
Moniz [41]	20 (65)	44.3, 14.8	MDD non suicide attempters	17.2, 7.3 (HAM-D); 2.3, 1.0 (BSI-D)		20 (65)	43.3, 14.9	–		Go/No-Go Task, ToL, Victoria Stroop Test, WCST, Finger Tapping Task, TMT, Verbal Fluency Test, AVLT		D < HC: processing speed, cognitive flexibility, motor speed, planning, inhibition, EF
Neu [42]	67 (67.2)	51.7, 12.0	MDD, IP	19.1, 4.7 (MES)		63 (69.8)	52.4, 11.3	–		RAVLT, TMT A, verbal fluency, WMS-R (Subscale Visual Memory)		D < HC: verbal learning and memory, processing speed, verbal fluency, visual memory
Reppermund [47]	53 (52.3)	43.5, 8.0	MDD, IP	25.1, 5.1 (HAMD)		13 (53.8)	46.4, 9.5	–		TAP: Alertness + Divided Attention, ZVT, Aufmerksamkeits-Belastungstest d2, WMS, verbal fluency tasks, SPM, CANTAB		D < HC: verbal learning and memory, WM, attention, processing speed, EF
Schmid [50]	30 (47)	26.2, 5.9	FE MDD, OP	24.6, 3.7 (MADRS)		30 (47)	26.2, 5.7	–		D-KEFS: Colour-Word Interference, verbal fluency CWIT, TMT, Tower Test		D < HC: inhibition, semantic fluency D = HC: mental flexibility, phonemic fluency, planning, problem solving
Schulze [51]	34 (59)	26.2, 5.9	Moderate depressive disorder OP	–		76 (59)	24.9, 5.7	–		MWT-A, LPS-3, ToH, WCST, TAP: WM, attention, CPT		D < HC: working speed D = HC: set shifting, planning, inhibition, WM, sustained attention
Schwert [52]	103 (69)	42.8, 13.02	Acute recurrent MDD, OP	18.1, 5.5 (HAMD-17); 26.6, 9.7 (BDI-II)		103 (69)	42.7, 12.5	–		COGBAT: TMT A + B, Alertness, Divided Attention, N-back verbal, Figuraler Gedächtnis Test, Go-NoGo, ToL		D < HC: processing speed, divided attention, verbal WM, figural memory, inhibition D = HC: alertness, cognitive flexibility, planning
Taconnat [54]	21 (71)	29.7, 5.5	MDD, IP	11.8, 3.4 (HADS, Depression)		24 (67)	28.5, 4.6	7.1, 3.3 (HADS depression)		WCST, letter-comparison test, COWA: categorical fluency		D < HC: EF, cognitive speed, categorical fluency
Zaremba [57]	106 (60)	37.7, 13.3	MDD IP and OP	14.64, 4.25 (HDRS-17)		120 (56)	37.4, 13.5	1.40, 1.68 (HDRS-17)		WAIS-R: digit symbol substitution test, TMT A, RAVLT, WMS: Spatial Span, Letter-Number Sequences		D < HC: processing speed, verbal learning and memory, visuospatial learning and memory D = HC: WM

*MDD* Major Depressive Disorder, *CD* Currently depressed, *IP* inpatients, *OP* outpatients, *FE* first episode, *D < HC* significant differences in favor of HC, *D = HC* no significant differences, *p* < .05, *EF* executive Functions, *WM* working memory

*AVLT* Auditory Verbal Learning Test, *BDI* Beck Depression Inventory, *CalCAP* California Computerized Assessment Package, *CANTAB* Cambridge Neuropsychological Test Automated Battery, *COGBAT* Cognitive Basic Assessment, *COWA* Controlled Oral Word Association, *CPT* Continuous Performance Task, *D-KEFS* Delis-Kaplan Executive Function System, *HADS* Hospital Anxiety and Depression Scale, *HAMD/HDRS* Hamilton Depression Rating Scale, *LPS* Leistungsprüfsystem, *MADRS* Montgomery-Asberg Depression Rating Scale, *MMSE* Mini-Mental Status Examination, *MWT* Mehrfachwahl-Wortschatz-Intelligenz-Test, *PASAT* Paced Auditory Serial Addition Test, *RAVLT* Rey Auditory Verbal Learning Test, *RBANS* Repeatable Battery for the Assessment of Neuropsychological Status, *SPM* Raven’s Standard Progressive Matrices, *TAP* Testbatterie zur Aufmerksamkeitsprüfung, *TEA* Test of Everyday Attention, *TMT* Trail Making Test, *ToL* Tower of London, *ToH* Tower of Hanoi, *WAIS* Wechsler Adult Intelligence Scale, *WCST* Wisconsin Card Sorting Test, *WMS* Wechsler Memory Scale, *ZVT* Zahlenverbindungstest

### Attention

A slight majority of studies investigating attention found a significantly reduced performance for depressed patients. Deficits were predominantly apparent for alertness [22, 27, 37]. Schwert [52] also found a reduced alertness on a non-significant level (*p* = 0.067). Additionally, divided attention [52], sustained attention [20], selective attention [30], and attention switching [35] was significantly impaired. Reppermund et al. [47] stated that there are significant deficits in attention and showed a reduced performance in the depressed sample, but withheld results compared to the HC group. Two other studies’ results regarding the RBANS domain attention were inconsistent [18, 31]. Unimpaired performance was shown in attention switching [39] sustained attention [38, 51], and in attention span (WAIS digit span forward) [27, 35].

### Verbal and visual learning and memory

Significant differences between HC and CD patients are mainly found in verbal learning and memory [22, 24, 42, 47, 57] and in visual learning and memory [20, 22, 24, 35, 42, 52, 57]. Jia [31] showed that first episode drug-naïve depressive patients had deficits in delayed, but not in immediate memory, whereas medicated depressive patients presented deficits in immediate and delayed memory. By contrast, Baune [18] found differences just in the immediate memory (RBANS). Three other authors failed to show differences in visual memory [16], verbal and visual memory [39], and immediate and delayed visual memory [38].

### Visuospatial

In the domain visuoconstruction, two studies [18, 31] showed ambiguous results using the RBANS to examine the drawing of a geometrical figure and the organizing of lines according to their angles.

### Working memory (WM)

There is a similar amount of evidence for deficits in WM [20, 22, 27, 35, 47, 52] and for an unimpaired performance [16, 36, 39, 51, 57]. Gruber [25] dedicated an entire study to investigating different facets of WM and showed significantly reduced scores for CD patients in verbal WM tasks requiring the articulatory rehearsal mechanism controls. Additionally, CD patients scored worse, but no significant results were found in other WM tasks (non-articulatory maintenance of phonological information; visuospatial rehearsal/pattern maintenance).

### Verbal fluency

Most studies investigating language used categorical fluency tests to examine verbal semantic fluency. Significant differences in favor of the HC were shown in multiple studies [24, 35, 42, 54]. Schmid [50] also found differences in semantic fluency, but no differences in phonemic fluency or switching category. The same results were found in Hammar’s study [28] using the D-KEFS to examine multiple verbal fluency categories. Depressed patients had significantly worse results in semantic fluency, but not phonemic fluency or in a switching category condition. A similar trend is shown in Halvorsen et al.’s study [27] also through the D-KEFS: they found no differences in phonemic (*p* = 0.56) and switching category (*p* = 0.53), but a tendency towards a difference in semantic fluency (*p* = 0.1). Mak [39] could not find differences in verbal semantic fluency.

### Executive function

There is a similar amount of studies showing deficits in inhibition in CD patients [17, 28, 30, 41, 50, 52] and studies showing no deficits [35–37, 51]. While most studies report no significant differences in cognitive flexibility [27, 28, 30, 35, 36, 47, 50–52], two studies found differences [39, 41]. Planning did not differ between groups [28, 50–52] except in Moniz’s study [41]. For visual problem solving, there is evidence of deficits [47] and evidence of similar performance [50]. Albert [16] found no differences in EF measured by multiple tests, which included an assessment of cognitive flexibility. Also, Lyche [37] using the Attentional Network Test, found no significant deficits for the domain EF. Deficits for the depressed sample for EF was shown using the tasks “Stockings of Cambridge” (SOC) [38] and “intra-extradimensional set shift” [20]. The Wisconsin Card Sorting Test (WCST) also revealed deficits [41, 54].

## Remitted depressed (RD) patients vs. HC group

### Information processing speed

The majority of studies did not find a decrease of the information processing speed in RD patients [19, 26, 27, 30, 43, 57] (see Table 5). However, Halvorsen [27] showed a slower reaction in the RD sample. Preiss [45] showed deficits in the hospitalized sample but not in the non-hospitalized one. Differences between the groups were found in four studies [29, 34, 44, 56].

**Table 5:** Characteristics and main results of studies investigating remitted depressed samples

	Remitted depressed group				Healthy control group				
Author and year	*n* (% female)	Age	sample	Symptom severity	*n* (female)	Age	Symptom severity	Cognitive tests	Main results
Baune [18]	44 (missing /wrong)	44.2, 15.9	previous MDD	6.8, 4.3 (HAMD-D 17)	206 (61.2)	47.5, 15.2	N/A	RBANS	RD < HC: immediate memory, attention, RD = HC: visuospatial/construction Language Delayed memory
Bhardway [19]	20 (10.0)	34.3, 8.2	Recovered	3.5, 2.0 (HDRS)	20 (15.0)	33.0, 7.5	2.5, 2.0 (HDRS)	WCST, WAIS, MMSE, Vocabulary Test	RD < HC: planning, problem solving RD = HC: WM, visuomotor speed, shifting attention
Daniel [23]	25 (64.0)	50.6, 8.3	MDD remitted	3.2, 1.4 (HAM-D)	29 (62.1)	47.5, 12.6	N/A	MMSE, Babcock Story Recall Test, WCST, TMT-B, Stroop Color & Word, WAIS-R: Symbol-Number Association, Digit Span	RD < HC: EF, WM RD = HC: verbal memory
Grützner [26]	65 (80)	38.9, 14.3	Fully and partial remitted	8.75, 5.1 (HAMD), 15.56, 9.93 (BDI-II)	65 (80)	38.9, 14.2	1.85, 1.99 (HAMD); 2.62, 3.17 (BDI-II)	COGBAT: TMT A + B, Alertness, Divided Attention, Selective attention; Nback verbal; Figural Memory Test, CVLT; Go-NoGo-INHIB, ToL, WAIS: Symbol Coding Task;	RD < HC: attention, learning, memory, WM RD = HC: information processing speed, EF
Halvorsen [27]	81 (87.7)	37.4, 9.6	recovered MDD	7.7, 6.7 (BDI-II)	50 (78)	38, 12.7	3.1, 2.9 (BDI-II)	D-KEFS: Color-Word and Verbal Fluency, WCST, TMT A + B, calCAP, Halstead-Reitan Seashore Rhythm Test, WAIS-III: digit span forward, digit span backward, digit symbol coding	RD < HC: processing speed RD = HC: WM, EF, information processing, attention, verbal fluency
Hasselbalch [29]	88 (68)	59.8, 9.2	Remitted MDD	2.8, 2.4 (HDRS-17)	50 (70)	59.7, 8	1.7, 1.7 (HDRS-17)	TMT A + B, Symbol Digit Modalities Test, RAVLT, CCR, RCFT, Familiar Faces, Boston Naming Test, phonological fluency test, semantic fluency test, Stroop test incongruent/ interference, WCST, Letter-Number-Sequencing	RD < HC: attention, visuomotor speed RD = HC: memory, verbal function, EF
Hsu [30]	30 (66.7)	23.9, 6.3	Formerly depressed	7.6, 7.2 (BDI-II)	29 (62)	24.1, 6.8	5.9, 7.9 (BDI-II)	D-KEFS: Color-Word interference test, TMT A + B, Emotional Stroop Task	RD = HC: selective attention, inhibition, psychomotor speed, cognitive flexibility
Leposavic [34]	?	48.3, 7.8	Endogenous depression, remit-ed, IP	7.3, 1.5 (BDI); 6.0, 2.2 (HRSD)	?	47.7, 6.6	N/A	MMSE, TMT A + B, RCFT RAVLT, WCST	RD < HC: processing speed, attention switching, visual and verbal memory, WM, prolonged attention
Peters [43]	62 (72.3)	20.9, 1.6	Remitted MDD youth OP	2.7, 3.4 (HAM-D)	43 (57.5)	20.7 (1.7)	0.4, 1.0 (HAMD)	Stroop Color and Word Test, COWAT, WAIS-IV: Digit Symbol, TMT A + B, Parametric Go/NoGo Task	RD < HC: cognitive control RD = HC: verbal fluency, processing speed, Conceptual reasoning & set shifting, processing speed with interference resolution
Preiss [44]	97 (52.6)	46.3, 12.0	RD	11.8, 7.0 (BDI-II), 4.4, 3.0 (MADRS)	97 (52.6)	46.1, 12.8	6.6, 6.0 (BDI-II)	AVLT, TMT A + B	RD < HC: verbal learning and memory, processing speed, cognitive flexibility
Preiss [45]	46 (45.7)	47.3, 10.4	RD, previously hospitalized	11.8, 6.9 (BDI-II), 4.3, 3.0 (MADRS)	92 (54.3)	46.2, 12.0	6.3 (5.7) (BDI-II)	AVLT, TMT A + B	RD < HC: delayed recall, processing speed RD = HC: learning, EF
Preiss [45]	46 (63)	43.5, 13.0	RD, non- hospitalized	11.3, 7.2 (BDI-II), 4.5, 3.2 (MADRS)	92 (54.3)	46.2, 12.0	6.3 (5.7) (BDI-II)	AVLT, TMT A + B	RD < HC: delayed recall, RD = HC: Learning, EF, processing speed
Wekking [56]	137 (75)	44.9, 9.4	Remitted MDD	3.7, 2.9 (HDRS)		Normative data		Stroop Color-Word Test; Memory Comparison Task; Digit Span; Rivermead Behavioral Memory Tests: Story Recall, Dutch CVLT	RD < HC: processing speed, WM, verbal memory RD = HC: EF
Zaremba [57]	119 (67)	38.5, 13.9	Remitted MDD OP	2.60, 2.23 (HDRS-17)	120 (56)	37.4, 13.5	1.40, 1.68 (HDRS-17)	WAIS-R: Digit symbol substitution test, TMT A, RAVLT, WMS, WAIS-3: Letter-Number Sequences	RD = HC: processing speed, verbal learning and memory, visuospatial learning and memory, WM

*MDD* Major Depressive Disorder, *RD* Remitted depressed, *IP* inpatients, *OP* outpatients, *FE* first episode, *D < HC* significant differences in favor of HC, *D = HC* no significant differences, *p* < 0.05, *EF* executive Functions, *WM* working memory. *AVLT* Auditory Verbal Learning Test, *BDI* Beck Depression Inventory, *CalCAP* California Computerized Assessment Package, *CCR* Category Cued Recall, *COGBAT* Cognitive Basic Assessment, *COWA* Controlled Oral Word Association, *CVLT* California Verbal Learning Test, *D-KEFS* Delis-Kaplan Executive Function System, *HADS* Hospital Anxiety and Depression Scale, *HAMD/HDRS* Hamilton Depression Rating Scale, *MADRS* Montgomery-Asberg Depression Rating Scale, *MMSE* Mini-Mental Status Examination, *RAVLT* Rey Auditory Verbal Learning Test, *RBANS* Repeatable Battery for the Assessment of Neuropsychological Status, *RCFT* Rey-Osterrieth Complex Figure Test, *TMT* Trail Making Test, *ToL* Tower of London, *WAIS* Wechsler Adult Intelligence Scale, *WCST* Wisconsin Card Sorting Test, *WMS* Wechsler Memory Scale

### Attention

Deficits in attention were observed in various tests [18, 26, 29, 34], while several other studies showed comparable performances in shifting attention [19] and selective attention [30] or in general attention [27].

### Verbal and visual learning and memory

Significant deficits in RD patients were found in visual and verbal memory [26, 34, 44], in verbal immediate and delayed recall [56], and in just the immediate memory [18]. Slightly different results were found in Daniel’s study [23] in verbal memory, *p* = 0.05; the difference did not reach significance. Preiss [45] revealed deficits in the delayed recall but not in learning. Other studies showed no differences in delayed memory [18] and verbal and nonverbal memory [29]. Similarly, Zaremba [57] showed unimpaired performance in verbal and visual learning and memory.

### Visuospatial

Comparable results are shown for both groups by one study [18].

### Working memory

For WM the results are relatively inconsistent. Some studies demonstrated significant differences [23, 26, 34, 56], while others did not [19, 27, 57].

### Verbal fluency

When testing for language, particularly for verbal fluency, no differences between the two groups were reported [27, 29, 43].

### Executive functions

No differences were found for inhibition [26, 30]. Most studies showed no deficits in cognitive flexibility assessed by the TMT-B [26, 30, 45] or a verbal fluency task with a switching condition [27]. One study reported impairment in the RD group [44]. For planning, two studies reported no differences [26, 29], one reported differences [19]. Bhardway [19] pointed out reduced problem solving. Peters [43] demonstrated differences in cognitive control (Go/NoGo Task), but revealed similar performance between the groups in set shifting and conceptual reasoning. Daniel [23] showed deficits in a general EF factor, contrary to multiple other studies [27, 29, 56].

For a summarized overview for currently and remitted depressed vs. healthy control groups please see Table 6.

**Table 6:** Summary of main results for currently and remitted depressed vs. healthy control groups

	Currently depressed vs. HC	Remitted depressed vs. HC
D < < < HC	Information processing speed, verbal learning and memory, visual learning and memory	–
D < < HC	Attention, inhibition, verbal fluency, WM	Attention, verbal learning and memory, visual learning and memory, WM
D < HC	–	Information processing speed, planning
D = HC	Cognitive flexibility, planning	Cognitive flexibility, verbal fluency

Notes A cognitive domain was included if it was investigated by at least 3 studies. HC: healthy control group

<  <  < : strong tendency (more than 75% of studies report deficits for depressed group)

<  < : moderate tendency (more than 50% of studies report deficits for depressed group)

< : weak tendency (more than 25% of studies report deficits for depressed group)

= : similar (25% or less of studies report deficits for depressed group)

## CD vs. RD patients

Baune [18] showed deficits in the domains attention and construction in favor of the RD group (RBANS). No differences were found for immediate and delayed memory and for language.

Halvorsen [27] found deficits exclusively in the acute phase for WM. Other domains did not show any further discrepancies.

## Follow up studies

In a follow up study, Ardal and Hammar [17] investigated whether depressive symptoms persist after recovery of a recurrent depressive episode. Using the Stroop test for cognitive inhibition, they tested a sample of 38 participants in the acute phase, 6 months later, and after recovery 10 years later. They found a significant difference between the depressed group and the HC in all three stages, indicating that neuropsychological impairment in the acute phase persists over the lifetime.

In another follow-up study, Boeker [20] showed that despite clinical recovery through assessment of depression severity no improvements were found in WM, EF, and sustained attention. However, better results were shown by RD patients in learning and memory.

In a sample of 79 depressed patients, Roca [48] found that after six months, RD patients were significantly better than non-remitted in processing speed, WM, selective attention/response inhibition, planning, verbal fluency, and especially in set-shifting. No improvement was shown for cognitive inhibition.

## First episode depressed vs. recurrent depressed

In-patients with a first episode (*n* = 50) or a recurrent depressive disorder (*n* = 160, average of 4.4 episodes) were tested in Talarowska’s study [55]. The depressive ratings were similar. Significantly better results in favor of the first episode group were found for information processing speed, learning, visual and verbal memory, WM, EF, and verbal fluency. This tendency was already visible when comparing patients with one episode with others with two episodes.

Kaygusuz [32] examined differences between first episode and recurrent depression. The recurrent depression group was more educated than the first episode group and no differences between the two groups were apparent.

## Severity of depression

Multiple studies reported a negative correlation of neuropsychological functioning with depression severity. Talarowska [55] revealed a negative correlation between symptom severity and performance regarding information processing speed, learning, verbal memory, EF, and verbal fluency. Boeker [20] found an association of depression severity and learning and memory. Likewise, Kaygusuz [32] showed a negative correlation of attention, encoding, learning, naming, and mental speed with depression severity after dividing the sample in mild and severe depression. Schwert [52] showed that the severity of MDD predicted significantly worse results in planning and divided attention. Finally, Liu [35] found that depression severity correlated with decreased WM performance.

Contrary results were published by Reppermund [47] who tested 25 facets of cognitive performance and only found one correlation (WM) with depression severity. In further studies, no association between depression severity and global cognitive functioning or verbal or figural memory [40] and cognitive performance [50] was reported.

## Additional comparisons

Five studies summarized the frequency of cognitive impairment. All of the following studies except the last one define scores lower than the 16th percentile–one standard deviation below the mean of the control group – as impaired.

The McClintock [40] study focused on the frequency of cognitive dysfunction and on correlations with symptom severity of a severely unipolar depressed group of patients referred for ECT. The authors found out that 41% of the MDD group were impaired in the MMSE, 29% were impaired in verbal memory, and 52% were impaired in visual memory.

Schwert [52] found impairment rates for acute unipolar depression of 77% in attention, 57% in EF, 39% in figural memory, 31% in WM, and 23% in processing speed. While 52% of the HC group showed no cognitive impairment at all, cognitive impairment was observed at 26% in the MDD group. 2% of the CD group were impaired in all five tested domains (0% in the HC).

In Reppermund’s study [47], the highest rates of impairment for patients in acute depression were found in tasks involving EF (60%) and alertness (57%). At discharge, when 43 of 53 patients were considered remitted, significant improvement was found in 10 of the 25 test scores. For example, impairment was still found in EF (57%), alertness (40%), and divided attention (47%).

In a sample of RD patients, Preiss [45] found that 34% of the former hospitalized and 20% of the never hospitalized patients presented cognitive deficits.

Defining scores lower than the 5th percentile (1.63 SD below the mean) as cognitive impairment, Wekking [56] investigated remitted, former depressed patients. Of this sample the highest impairment was found for different assessments of memory (9–34%) and for speed of memory processing (13–28%). The impairment found for WM was 13–15%, for speed of information processing 11–16%, and for EF 2%.

Comparing groups of CD patients with depressed BD-I patients and euthymic remitted BD-I patients, Maalouf [38] revealed that just the MDD and BD depressed group were impaired on EF, but not the BD euthymic patients. In Daniel’s study [23] RD and remitted BD-I patients were compared. The performances of neither the EF nor the WM nor the verbal memory differentiated. Similar to these findings, Liu [35] did not find any differences using an extensive test battery on patients in an acute depressive state with either the diagnosis depression or BD-II. Testing another sample with treatment naïve MDD and BD-II patients in an acute depressive state, Mak [39] found significantly slower psychomotor speed for the MDD group. Like the previous studies, no differences were shown in learning and memory, frontal EF, and verbal fluency.

When compared to patients with SCH, Schaub [49] revealed significantly better results for MDD in the domains verbal and visual short-term memory, verbal fluency, visual-motor coordination, information processing, and selective attention. Practical reasoning, general verbal abstraction, spatial-figural functioning, and speed of cognitive processing did not differ. Conducting a larger-scaled study with 102 patients with a diagnosis of depression without psychotic features and 72 patients with SCH, Gooren [24] demonstrated an overlap between the two disorders: patients with unipolar depression revealed better results in verbal fluency and visual memory, verbal learning and processing speed were comparable. The better result in delayed memory in SCH could be due to better learning. Sostaric [53] also pointed out a certain overlap regarding cognitive performance in MDD and SCH, with the sample of hospitalized patients in both groups showing significant lower scores in information processing speed, shifting of attention, and in visual and verbal learning and memory compared to normative data. The SCH group achieved significantly better results in WM and in the visual delayed recall.

Castaneda [21] investigated two groups of either pure MDD patients or comorbid MDD patients, with mostly anxiety disorders and substance abuse/dependence. No statistically significant differences were found in verbal and visual short-term memory, verbal long-term memory and learning, attention, processing speed, and EF.

The comparison of neuropsychological deficits in suicide attempters and non-attempters with a history of unipolar or bipolar depression revealed that past suicide attempters performed significantly worse in attention, memory, and WM [33]. Suicide attempters also achieved worse, but non-significant differences in learning, language fluency, and impulse control. Moniz [41] showed poorer cognitive inhibition in suicide attempters compared to non-attempters. However, the suicide attempters presented better results in planning.

Constant [22] compared the cognitive performance between participants with chronic fatigue syndrome and MDD with results showing similar reaction times as well as performance on memory tasks and alertness. For WM, depressed participants showed a worse performance than participants with chronic fatigue syndrome.

Leposavić [34] investigated hospitalized depressed vs. demented patients. Depressed patients showed significantly better performances in processing speed, attention switching, visual and verbal memory, WM, and prolonged attention.

To compare patients with MDD and OCD, Rampacher [46] matched the groups according to depression severity. They found significant differences in visual organization and problem solving in favor of MDD. No significant differences could be shown for verbal and visual memory, delayed visual response, visuo-motor speed/set shifting, and verbal fluency.

## Discussion

As previous research suggests a broad range of deficits in Major Depressive Disorder, we aimed to update the available evidence and systematically review studies published between 2009 and 2019, investigating cognitive impairment in adult depressive patients in the acute and remitted state. Additionally, we assessed a possible risk of bias of the included primary studies and compared the neuropsychological profiles of depressive patients to those suffering from BD or SCH.

The majority of included studies focused on an experimental design with a CD and a HC group. Large differences in favor of the control group were found in information processing speed with 12 out of 15 studies reporting significant differences using mainly the TMT-A. Besides that, strong tendencies were found for deficits in verbal and visual learning and memory. For verbal fluency, moderate tendencies for deficits were shown for semantic tests, but not for phonemic or for category switching tasks. Nine out of 15 studies revealed deficits in attention, mainly a reduced alertness. Sustained attention and attention switching remained unclear. Additionally, inhibition and WM deficits were reported in more of 50% of studies testing these areas. For WM, tasks that require the articulatory rehearsal mechanism controls seem to be especially affected. Studies testing for EF in broader domain mainly showed deficits. For cognitive flexibility, two out of 11 and for planning one out of five studies showed significant differences. With respect to visuospatial skills and visual problem solving, one study supports deficits in these domains while another study contradicts these findings. For a summarized overview, please see Table 6. The strong tendency for deficits in information processing speed, learning and memory, and verbal fluency is in compliance with former summaries [4, 5, 48] and our hypothesis (1). EF which is usually described as one of the most impaired domains [4, 5] showed a weaker, but still moderate tendency for deficits in our systematic review of the current literature. This was mainly due to results on inhibition and WM. Other features of the broad field of EF, e.g., planning and cognitive flexibility assessed mainly through the TMT-B, do not seem to be impaired in CD patients.

Comparing samples of RD patients with HC, no clear tendency for deficits in the RD group was reported as it was shown for CD patients. The highest ratio of impaired function was found for visual learning and memory in three out of four studies. Other moderate tendencies were seen for verbal learning and memory, attention, and WM. Information processing speed was impaired in 40% of the studies. Notably, Preiss [45] showed deficits in a former hospitalized sample, but not in the non-hospitalized one. Planning was found to be deficient in one out of three studies. No differences were revealed for cognitive flexibility and verbal fluency. In addition, EF was not found to be impaired. Two studies focusing on the direct comparison between CD and RD found deficits for attention, construction, and WM in the acute sample. Follow up studies likewise suggest a persistence of cognitive impairment after remission. Ardal and Hammar [17] found ongoing deficits in cognitive inhibition and propose that cognitive inhibition could be an irreversible vulnerability marker. Also, Boeker [20] showed persisting deficits in EF, WM, and sustained attention suggesting these to be trait markers. However, impairment in visual learning and memory showed a significant increase after recovery and could be state marker according to the authors. In Roca’s study [48] improvements for the RD sample were revealed in most domains. Cognitive inhibition was found to be impaired like in Ardal’s study [17]. The heterogenous results between Boeker [20] and Roca [48] could be due to defining the “recovered” depressed sample. Boeker’s recovered group shows a mean of 10.5 (SD: 8) in the HDRS 21 while Roca’s group had to score lower than 7 in the HDRS 17. Consequently, in Boeker’s “recovered” sample there is an inclusion of patients responding well to the treatment, but not remitted according to the ACNP Task Force [60]. These findings line up with previous studies and our hypothesis (2) that just a partial cognitive improvement is achieved in remission.

The included studies revealed a greater cognitive impairment in patients suffering from recurrent episodes than in first episode depressed patients. Severity of depression was found to have a positive correlation with cognitive impairment in five out of eight studies. For the most part, more dominant deficits were found for learning, memory, attention, processing speed, WM, and EF. This confirms our hypotheses (3, 4) and lines up with prior research [9, 10]. It is possible that the prescription of medication might have narrowed down the span of reported depressive symptoms consequently leading to false identification of severely depressed patients as just moderately depressed. Mixing up the groups would end up obscuring possible greater differences. We cannot rule out that additional treatment constitutes a factor in the more impaired group. However, in the review process, insufficient data could be collected on, for example, medication.

Most studies investigating differences of the cognitive pattern for depression and BD showed similar results if the groups were in an equal state. Few differences were found in samples of euthymic or depressed patients with either diagnosis. These results align with current research demonstrating no worse performance by BD samples [61, 62] as well as contradicts other current research [63, 64].

Three of the included studies which investigated differences between MDD and SCH suggested a partial overlap in deficits. Besides a partial overlap, a heterogeneity in results was observed, likely due to the inclusion of different subtypes of SCH, for example, overrepresentation of the better performing paranoid subtype [65], the mostly uncontrolled influence of medication, and other factors.

Comorbidity with mostly anxiety disorders did not seem to affect cognitive performance. Former suicide attempters in general showed more cognitive deficits than non-attempters, with inhibition being one of the most evaluated factors. Comparisons of MDD with chronic fatigue syndrome showed overall similar results. One study found significantly better results for depressed patients compared with a demented sample. Patients with an obsessive–compulsive disorder performed significantly worse than depressive patients in visual organization and problem-solving tasks. Not enough studies reported on these comparisons to draw reasonable conclusions. Nevertheless, the studies emphasized that other diagnoses seem to impact the cognitive performance in a different, often more impairing way.

Our review revealed that just some studies reported frequencies of cognitive impairment and those that did reported a broad range of frequencies. Based on the assumption that impairment is defined by one SD under the mean of the control group, one study found that 74% of the CD showed deficits compared to up to 34% of RD. In the acute phase, frequencies of 57–77% in attention, 57–60% in EF, and 29–52% in memory were seen. At discharge, Reppermund [47] discovered an impairment of 57% in EF and of 40% in alertness. Memory functions were shown as being one of the most impaired cognitions. Surprisingly, when investigating the frequency of cognitive deficits in patients in the acute state of depression, only around 30–50% were affected by memory deficits. A possible explanation could be a high variability in the extent of memory deficits, leading nonetheless to differences in mean comparisons.

Overall, the presented results for the acute phase do not consistently support the general cognitive effort hypothesis, which states that automatic processes are normal but that tasks requiring effortful processing are impaired [66]. Likewise, the included studies did not show specific impairment in memory or EF in the acute phase of depression [67]. The evidence instead speaks in favor of the global-diffuse hypothesis, which expects an extensive reduced cognitive performance in multiple areas [69] with underlying impairment in attentional processes. Impairment in attention was present in up to 77% of the current sample of studies, even more severe than EF. In line with this, multiple review articles [70, 71] on neuroimaging studies suggest that attentional deficits in major depression are accompanied by reduced connectivity within frontoparietal control systems, as well as imbalanced connectivity between control systems and networks involved in internal or external attention [70]. This leads to a favoring of internal thoughts at the cost of engaging with the external world in depression, and may partially explain the well-documented bias towards rumination, as well as the global cognitive deficits, as summarized in the current review.

Moreover, our results support the common pathway disorder hypothesis. Supporters of this hypothesis see the global deficit based on impaired functional networks with attentional and executive elements common in different diagnoses [72, 73]. Persistent impairment after remission points to sustained neurocognitive deficits rather than a state character of these impairments. However, the development of neurocognitive impairments over time should be examined in more detail: studies examining neurocognitive functions prior to the first MDD episode and with long-term follow-up provide tentative evidence for a progressive decline in neurocognitive functioning [74].

## Study limitations and recommendations for future research

Evaluating the risk of bias across studies leads to an unclear risk in our study set. Therefore, the current results should be interpreted with caution. The vast majority of studies with a high risk of bias showed limitations in the selection process. Therefore, the current results are limited due primarily to methodological deficits in the selection of clinical groups and HC groups (i.e. no correction for differences between groups concerning age, gender, or educational level). Furthermore, most studies did not quantify the duration and number of episodes of depression in clinical groups, did not consider putative interaction effects between medication and cognitive performance in clinical groups, and paid little attention to comorbid psychiatric or neurological illnesses that could moderate the correlation between depression severity and cognitive deficits. Furthermore, the matching of clinical and healthy groups was limited, particularly concerning the estimation of premorbid cognitive performance levels of clinical patients with those of the HC group. Despite common reporting of education levels, an explicit assessment of premorbid intelligence was only conducted by 48% of the studies. Concerning the blinding procedure, only four out of 42 studies adequately encountered a possible detection bias by blinding assessors. Conducting a meta-analysis could be an option for future research. We, however, favored a systematic review approach because of a broader, and therefore, more heterogeneous range of hypotheses.

Given that one quarter of the included studies bear a high risk of bias according to our risk assessment, adherence to a standardized methodology is essential for future studies, especially concerning the selection. In Table 7 we provide five practical recommendations for future researchers.

**Table 7:** Five practical recommendations to lower the risk of bias and to improve the quality of neuropsychological research

1. Match clinical and control groups based on premorbid intelligence measurements of the patients (e.g. National adult reading test, NART) as well as age, gender, and educational level
2. Control for moderating effects of medication on the correlation between depression symptom severity and cognitive deficits 3. Improve the characterization of clinical groups concerning the duration and number of depressed episodes, as well as putative comorbid psychiatric or neurological illnesses that may impact on cognitive performance
4. Neatly execute blinding procedures of assessors to avoid an overestimation of differences between clinical and healthy groups
5. Apply a generally used cut-off when reporting clinically significant cognitive impairments (e.g. one standard deviation below the mean)

## Conclusions

Current studies about CD patients reveal strong support for deficits in processing speed, learning and memory, and impairment in attention, inhibition, verbal fluency, and WM. Despite remission of the depressed syndrome, evidence for persistent deficits in attention, learning and memory, and WM is reported. Nevertheless, RD patients show smaller deficits than in acute state, as shown in direct comparisons and in follow-up studies. Evidence for a positive correlation between number of episodes and cognitive deficits and as well as between depression severity and cognitive deficits is reported. Most studies did not find differences in the cognitive profiles of patients with MDD and BD I or II. For a comparison with SCH heterogeneous results were reported, partially suggesting an overlap of the cognitive profiles. Specific studies are needed for a further understanding of differences in the cognitive profiles between depression and other disorders. Attentional deficits were found in up to 77% of acute MDD patients. The results support the assumption of global deficits and the final common pathway disorder hypothesis for cognitive dysfunction in patients suffering from MDD. Due to an unclear risk of bias across our study set, these results should be interpreted cautiously. Based on our risk of bias assessment, we derive recommendations for future research to lower the risk of bias and to improve the quality of neuropsychological research.

## Data Availability

Supplemental data are available for open access at https://osf.io/hn3w8/.
